# Optimization of a laparoscopic procedure for advanced intrahepatic cholangiocarcinoma based on the concept of “waiting time”: a preliminary report

**DOI:** 10.1186/s12885-022-10323-x

**Published:** 2022-11-28

**Authors:** Cheng-Yu Liao, Dan-Feng Wang, Bin-Hua Jiang, Long Huang, Tian-Sheng Lin, Fu-Nan Qiu, Song-Qiang Zhou, Yao-Dong Wang, Xiao-Chun Zheng, Yi-Feng Tian, Shi Chen

**Affiliations:** 1 Shengli Clinical Medical College of Fujian Medical University, Fuzhou, China; 2 Department of Hepatobiliary Pancreatic Surgery, Fujian Provincial Hospital, Fuzhou, 350001 China; 3 Department of Anesthesiology, Fujian Provincial Hospital, Fuzhou, 350001 China; 4 Fujian Emergency Medical Center, Fujian Emergency Medical Center, Fujian Provincial Key Laboratory of Emergency Medicine, Fujian Provincial Key Laboratory of Critical Medicine, Fujian Provincial Coconstructed Laboratory of “Belt and Road”, Fuzhou, China

**Keywords:** Intrahepatic cholangiocarcinoma, Laparoscopic, Lymph node dissection, Optimization, Hemihepatectomy

## Abstract

**Introduction:**

Clinicians increasingly perform laparoscopic surgery for intrahepatic cholangiocarcinoma (ICC). However, this surgery can be difficult in patients with advanced-stage ICC because of the complicated procedures and difficulty in achieving high-quality results. We compared the effects of a three-step optimized procedure with a traditional procedure for patients with advanced-stage ICC.

**Methods:**

Forty-two patients with advanced-stage ICC who received optimized laparoscopic hemihepatectomy with lymph node dissection (LND, optimized group) and 84 propensity score-matched patients who received traditional laparoscopic hemihepatectomy plus LND (traditional group) were analyzed. Surgical quality, disease-free survival (DFS), and overall survival (OS) were compared.

**Results:**

The optimized group had a lower surgical bleeding score (*P* = 0.038) and a higher surgeon satisfaction score (*P* = 0.001). Blood loss during hepatectomy was less in the optimized group (190 *vs.* 295 mL, *P* < 0.001). The optimized group had more harvested LNs (12.0 *vs.* 8.0, *P* < 0.001) and more positive LNs (8.0 *vs.* 5.0, *P* < 0.001), and a similar rate of adequate LND (88.1% *vs.* 77.4%, *P* = 0.149). The optimized group had longer median DFS (9.0 *vs.* 7.0 months, *P* = 0.018) and median OS (15.0 *vs.* 13.0 months, *P* = 0.046). In addition, the optimized group also had a shorter total operation time (*P* = 0.001), shorter liver resection time (*P* = 0.001), shorter LND time (*P* < 0.001), shorter hospital stay (*P* < 0.001), and lower incidence of total morbidities (14.3% *vs.* 36.9%, *P* = 0.009).

**Conclusions:**

Our optimization of a three-step laparoscopic procedure for advanced ICC was feasible, improved the quality of liver resection and LND, prolonged survival, and led to better intraoperative and postoperative outcomes.

## Introduction

Intrahepatic cholangiocarcinoma (ICC) is the second-most common primary liver malignancy. At diagnosis, ICC is often at an advanced stage and is accompanied by lymph node (LN) metastasis. In this case, hepatectomy is the only potential cure [1–5]. In contrast to hepatocellular carcinoma (HCC), the most common liver malignancy, there is controversy regarding the routine use of LN dissection (LND) in patients who receive open surgery or laparoscopic surgery. Many researchers believe that LND does not significantly improve overall survival (OS), but can lead to more accurate staging, reduce local recurrence, and provide information related to prognosis [6–9].

Several expert consensus statements recommend routine hepatoduodenal ligament dissection, especially for patients with advanced-stage ICC [4, 10–13]. Because it is relatively easy to perform dissection of the hepatoduodenal ligament LNs during traditional open surgery, most surgical procedures first remove the LNs and then perform liver resection. The low central venous pressure (LCVP) anesthesia technique allows the widespread use of laparoscopic surgery for LND in patients with ICC, because it provides more rapid recovery and it follows the same traditional process used during open surgery (“LND first”) [14–17]. However, patients with advanced-stage ICC often require resection of large liver volumes, such as integratedhemihepatectomy combined with ipsilateral caudate lobectomy and LND.

Patients with advanced-stage ICC have more affected LNs, and a laparoscopic approach for LND can be technically challenging because it requires much more time than an open approach to achieve fine 360° skeletalization of all vessels in the hepatic hilum. In this situation, the liver experiences a longer time of LND before hepatic parenchyma resection, which we define as the “waiting time” [10, 14, 18–21]. This inevitably leads to two major problems. First, when LND is performed before liver resection, it can lead to a long “waiting time”. In this case, a patient’s liver remains in a relatively ischemic or hypoxic state because of strict fluid restriction due to the prolonged LCVP before resection of hepatic parenchyma. This can cause microcirculation disorders and the accumulation of acidic substances, thus disrupting the condition of the hepatic surgical field and the ability to control LCVP [21–29]. Second, to perform liver resection as soon as possible, some surgeons may rush to complete the LND, which may reduce the quality of the procedure and potentially lead to incomplete or inadequate LND [10, 30–33].

Thus, to improve the condition of the hepatic surgical field and the quality of liver resection and laparoscopic LND, our center optimized the surgical procedure to reduce this “waiting time”. We modified the traditional laparoscopic procedure of LND followed by liver resection to a three-step sequential procedure that consists of pre-dissection of hepatic hilar vessels, liver resection, and then LND. This compared the quality of liver resection and LND, and the survival benefits of laparoscopic radical resection of advanced-stage ICC from the traditional procedure and our optimized procedure.

## Methods

### Patients

This prospective observational study examined patients who received laparoscopic radical liver resection for advanced-stage ICC by our optimized three-step procedure from January 2018 to March 2020. Data of patients who received traditional laparoscopic radical ICC surgery from January 2013 to December 2017 were retrospectively collected for comparison. This study was approved by the local Ethics Committee, and all procedures were performed in accordance with the 2013 Declaration of Helsinki. Written informed consent was provided by each patient.

The inclusion criteria were: (*i*) patient age of 18 to 80 years old; (*ii*) whole body PET-CT examination before the operation showing that the liver and duodenal ligament LNs had hyper-metabolism (suggesting LN metastasis) and no distant metastasis; (*iii*) pathological diagnosis of intrahepatic cholangiocarcinoma; (*iv*) receipt of laparoscopic radical left or right hemihepatectomy plus ipsilateral caudate lobectomy and hepatoduodenal ligament LND; and (*v*) valid and complete surgical video and perioperative data. The exclusion criteria were: (*i*) abdominal implant transfer or distant transfer (in which case only palliative surgery was indicated); (*ii*) co-occurrence of another malignant tumor; (*iii*) pathological results suggesting mixed hepatocellular-cholangiocarcinoma, hilar cholangiocarcinoma, or gallbladder cancer; and (*iv*) need for biliary anastomosis. All included patients were divided into an optimized group and a traditional group. All results are reported in line with the STROCSS guidelines [34].

### Operation procedures

All operations were performed by experienced surgeons who specialized in hepatobiliary surgery and had extensive experience in laparoscopic techniques. All patients were anesthetized using endotracheal intubation with a controlled LCVP strategy that required fluid restriction [35]. In particular, strict fluid restriction was used from the night before surgery until liver parenchyma resection, and the reverse-Trendelenburg position was used. Thus, most patients had CVPs close to 5 cmH_2_O, and some were even lower than 5 cmH_2_O. When it was close to the time for hepatic parenchyma resection, a small amount of nitroglycerin was used to reduce the CVP to below 5 cmH_2_O if necessary.

With the patient in the French position, a 5-hole method was used to establish a pneumoperitoneum and maintain a pneumoperitoneum pressure of 12 mmHg. First, the abdominal and pelvic cavities were examined to determine if there were metastatic nodules. After confirming there was no metastatic transplantation, an ultrasonic scalpel (Harmonic Scalpel; Ethicon, Cincinnati, OH) was used to free the ligamentum teres hepatis, falciform ligament, coronary ligament, and triangular ligament of the liver, and the gallbladder was then removed.

Patients in the traditional group received hepatoduodenal LND before resection of the liver (integrated left hemihepatectomy or right hemihepatectomy combined with ipsilateral caudate lobectomy). The sequence of procedures in the traditional group was: (*i*) LND (fine 360° skeletalization of all vessels in the hepatic hilum); (*ii*) ligation and cutting off the target side blood vessels and bile duct; and (*iii*) resection of target-side hepatic parenchyma. The sequence of procedures in the optimized group was: (*i*) pre-dissection of hepatic hilar vessels (simply freeing, ligating, and severing the target side vessels and bile duct for subsequent ligation and disconnection); (*ii*) resection of target-side hepatic parenchyma; and (*iii*) hepatoduodenal ligament LND (fine 360° skeletalization of the remaining vessels within the porta hepatis). All other procedures in the two groups were the same. The LND included station 12 (hepatoduodenal), station 8 (common hepatic artery), and station 13 (posterior to pancreas). For left-sided tumors, station 7 (left gastric artery) and station 1 (right esophageal crus) were also removed [36].

### Outcome measurements

#### Quality of liver resection

The condition of the hepatic surgical field was evaluated using a bleeding score and the surgeon’s satisfaction score [25, 37]. The bleeding score was 1 (minor bleeding, no aspiration required), 2 (minor bleeding, aspiration required), 3 (minor bleeding, frequent aspiration required), 4 (moderate bleeding, no visibility without aspiration), or 5 (severe bleeding, frequent aspiration required, very hard to perform surgery). The surgeon satisfaction score was 1 (bad), 2 (moderate), 3 (good), or 4 (excellent).

The total operation time, “waiting time”, liver resection time, LND time, total blood loss, blood loss during liver resection, and rate of transfusion and conversion were used to assess the quality of liver resection. All of these times were recorded by review of the operation video.

The “waiting time” was defined as the time from observation of the trocar puncture to liver parenchyma resection. The “waiting time” of the optimized group was the time needed to establish a laparoscopic tunnel plus the time needed for pre-dissection of the hepatic hilar vessels (simple separation and disconnection of the target side vessels and bile duct). The “waiting time” of traditional group was the time needed for establishment of laparoscopic tunnel, plus the time needed for fine 360° skeletalization of the hilar blood vessels, plus the time needed to sever the target side blood vessels and bile ducts.

#### Quality of LND

LND was evaluated using the pathological reports of LNs that were harvested from around the hepatoduodenal ligament. The number of harvested LNs, number of positive LNs, positive rate of LNs, and rate of adequate LND were used to assess the quality of LND. As described by the eighth edition of the American Joint Committee on Cancer (AJCC) guidelines for ICC, dissection was described as adequate when at least 6 nodes were harvested [10].

#### Other outcomes

Arterial blood gas analysis was used to measure lactate levels before anesthesia (preoperative lactate), immediately before liver resection (pre-resection lactate), immediately after removal of liver lesions (post-resection lactate), and before patient discharge from the postanesthesia care unit (postoperative lactate). The State Trait Anxiety Inventory (STAI) scale, which is routinely used in our center, was used to characterize the mental stress of the chief surgeon after the operation. The STAI measures stress using 6 items: 3 positive items (“I feel calm,” “I feel content,” and “I feel relaxed”) and 3 negative items (“I feel tense,” “I feel upset,” and “I feel worried”). Each item was self-rated on a 4-point scale, so the total score ranged from 6 (very low stress) to 24 (very high stress) [32]. Higher scores indicate higher stress. Postoperative outcomes, including recovery indicators and morbidities, were also recorded. The Clavein-Dindo classification was used to classify morbidities [38]. Bile leakage was diagnosed, classified, and treated as proposed by the International Study Group for Liver Surgery [39, 40]. The recovery indicators were postoperative length of stay (PLOS), time to resume out-of-bed activities, time for recovery of bowel movements, and time for recovery of oral intake of a semi-liquid diet.

### Follow up

Each patient received adjuvant chemotherapy and routine postoperative monitoring (liver function tests; routine blood examinations; serum CEA and CA19-9; and CT or MRI scans) every 3 months. Disease-free survival (DFS) and overall survival (OS) were calculated as the times from the operation to recurrence.

### Statistical analysis

All data were analyzed using SPSS version 26.0 (IBM Corporation, Armonk, NY). Propensity score (PS) matching (1:2) was performed using R software version 4.1.0 (“Matchit package”). Continuous variables were analyzed using Student’s *t*-test or the Mann–Whitney U test, as appropriate. Categorical variables were analyzed using Pearson’s *χ* ^*2*^ statistic or Fisher’s exact test. DFS and OS was analyzed using the Kaplan–Meier method and compared using the log-rank test. Logistic regression analysis was used to identify factors associated with morbidities, and Cox regression analysis was used to identify factors associated with DFS and OS. A *P* value below 0.05 was considered significant.

## Results

### Baseline characteristic

**Fig. 1:**
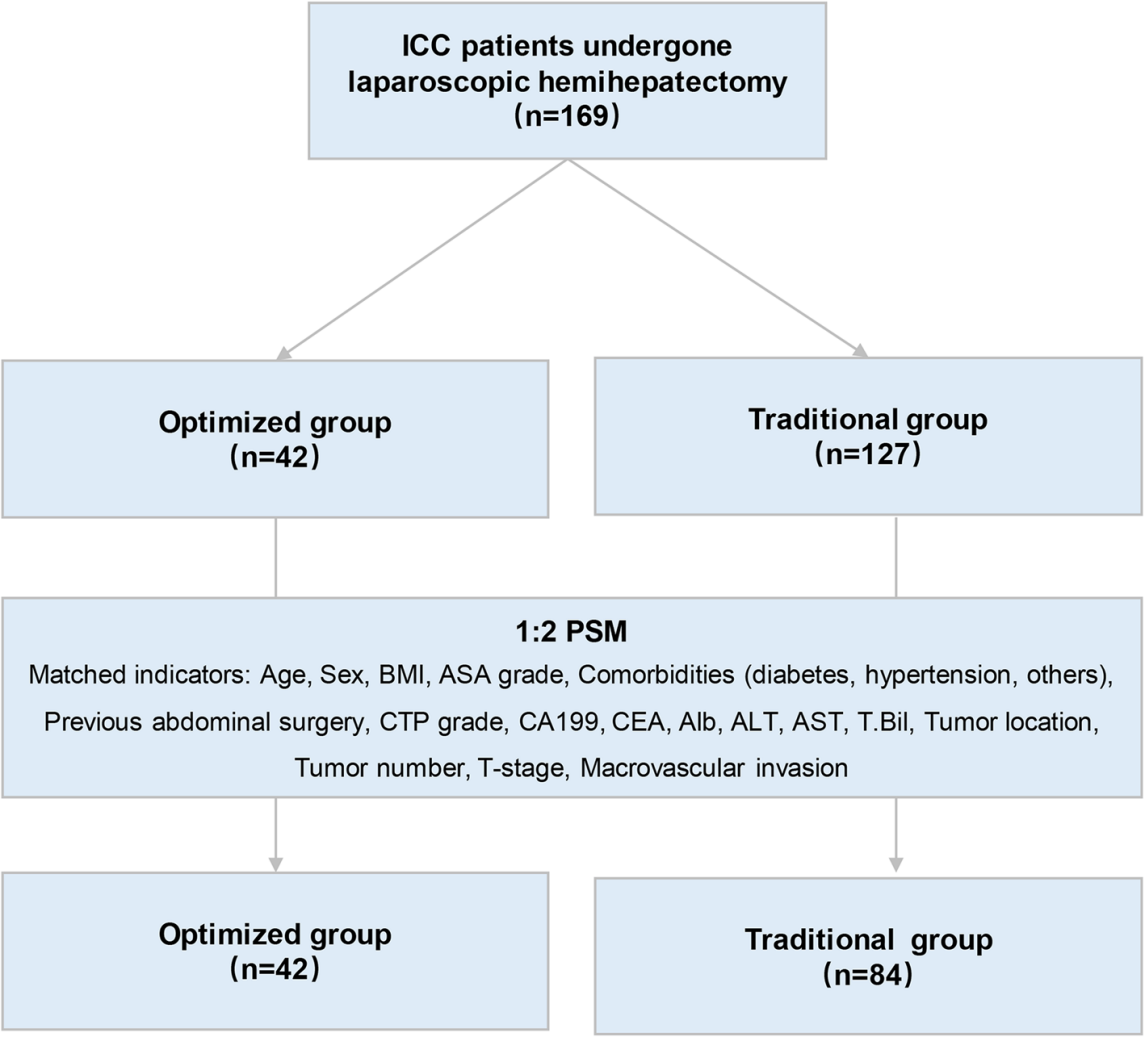
Identification of eligible patients and establishment of traditional and optimized groups by 1:2 propensity score matching. Abbreviations: ICC, intrahepatic cholangiocarcinoma; PSM, propensity score matching; BMI, body mass index; ASA, American Society of Anesthesiologists; ALB, albumin; ALT, alanine aminotransferase; AST, aspartate aminotransferase; T.Bil, total bilirubin

Before PS matching, 42 patients were in the optimized group and 127 patients were in the traditional group (Fig.  1).After 1:2 PS matching, there were 42 patients in the optimized group and 84 in the traditional group. There were also 24 males (57%) and 18 females (43%) in the optimized group and similar percentages (59.5% and 40.5%) in the traditional group (Table 1). Analysis of age, BMI, ASA score, comorbidities, serological indexes, tumor location, number of tumors, T stage, preoperative suspicious LN metastasis, and vascular invasion indicated no significant differences after matching (Table 1).

**Table 1:** Baseline characteristics

**Characteristics**	**Before PSM**			**After PSM**		
	**Optimized group**	**Traditional group**	***p***	**Optimized group**	**Traditional group**	***p***
	***n*** ** = 42**	***n*** ** = 127**		***n*** ** = 42**	***n*** ** = 84**	
Age, median[IQR], y	60.00(54.00–66.00)	63.00(55.50–67.50)	0.141	60.00(54.00–66.00)	61.00(54.50–66.00)	0.730
Sex, n (%)			0.751			0.798
Male	24(57.1)	69(54.3)		24(57.1)	50(59.5)	
Female	18(42.9)	58(45.7)		18(42.9)	34(40.5)	
BMI, median[IQR]	22.60(21.23–24.84)	23.83(21.68–26.04)	0.140	22.60(21.23–24.84)	23.48(21.23–26.19)	0.848
ASA class, n (%)			0.345			0.557
I	8(19.0)	16(12.6)		8(19.0)	10(11.9)	
II	19(45.2)	73(57.5)		19(45.2)	41(48.8)	
III	15(35.7)	38(29.9)		15(35.7)	33(39.3)	
Comorbidity, n (%)						
Diabetes mellitus	6(14.3)	22(17.3)	0.646	6(14.3)	11(13.1)	1.000
Hypertension	14(33.3)	44(34.6)	0.877	14(33.3)	27(27.3)	0.893
Others	5(11.9)	10(7.9)	0.303	5(11.9)	9(9.3)	1.000
Previous abdominal surgery, n (%)	3(7.1)	17(13.4)	0.213	3(7.1)	8(9.5)	0.750
Child–Pugh grade, n (%)			0.297			0.664
A	41(97.6)	119(93.7)		41(97.6)	80(95.2)	
B	1(2.4)	8(6.3)		1(2.4)	4(3.3)	
CEA, median[IQR], U/L	5.15(2.52–14.85)	4.34(2.53–11.20)	0.164	5.15(2.52–14.85)	4.34(2.54–11.20)	0.550
CA199, median[IQR], U/L	192.83(78.38–500.14)	95.30(29.74–475.00)	0.647	192.83(78.38–500.14)	102.95(32.04–578.20)	0.737
ALB, median[IQR], U/L	41.20(37.00–44.00)	43.00(39.00–46.00)	0.177	41.20(37.00–44.00)	42.00(38.25–45.60)	0.548
ALT, median[IQR], U/L	50.50(41.00–95.00)	42.00(24.00–72.00)	0.016	50.50(41.00–95.00)	47.50(28.00–76.00)	0.134
AST, median[IQR], U/L	36(23.00–62.00)	43.00(28.00–56.00)	0.327	36(23.00–62.00)	41.00(27.00–56.00)	0.469
T.Bil, median[IQR], U/L	17.00(14.30–24.60)	18.8(14.95–26.75)	0.791	17.00(14.30–24.60)	17.85(13.70–24.10)	0.770
Tumor location, n (%)			0.900			0.894
Left side	28(66.7)	86(50.9)		28(66.7)	55(65.5)	
Right side	14(33.3)	41(32.3)		14(33.3)	29(34.5)	
Tumor number, n (%)			0.395			1.000
Single	39(92.9)	121(95.3)		39(92.9)	79(94.0)	
Multiple	3(7.1)	6(4.7)		3(7.1)	5(6.0)	
T stage, n (%)			0.300			0.534
T1/T2	32(76.2)	86(67.7)		32(76.2)	68(81.0)	
T3/T4	10(23.8)	41(32.3)		10(23.8)	16(19.0)	
Macrovascular invasion, n (%)	9(21.4)	23(18.1)	0.634	9(21.4)	16(19.0)	0.752

The matched indicators were age, gender, body mass index (BMI), American Society of Anesthesiologists (ASA) classification, comorbidities, history of abdominal surgery, Child–Pugh classification, CEA, CA199, serum albumin (ALB), alanine aminotransferase (ALT), aspartate amino transferase (AST), total bilirubin (TBIL), tumor location, tumor number, T stage, and macrovascular invasion

*ICC* intrahepatic cholangiocarcinoma, *PSM* propensity score matching, *BMI* body mass index, *ASA* American Society of Anesthesiologists, *ALB* albumin, *ALT* alanine aminotransferase, *AST* aspartate aminotransferase, *T.Bil* total bilirubin

### Intraoperative outcomes

Twenty-eight patients in the optimized group received left hemihepatectomy and 14 received right hemihepatectomy; 56 patients in the traditional group received left hepatectomy and 28 received right hepatectomy. These differences were not significant (Table 2). Nine patients in the optimized group and 11 patients in the traditional group received vascular reconstruction (*P* = 0.752).

**Table 2:** Intraoperative and pathological outcomes

**Variates**	**Optimized group**	**Traditional group**	***p***
	***n*** ** = 42**	***n*** ** = 84**	
Surgery scope, n (%)			1.000
Left hemihepatectomy	28(66.7)	56(66.7)	
Right hemihepatectomy	14(33.3)	28(33.3)	
**The quality of liver resection**			
Total operative time, median[IQR], min	222(177–297)	280(250–357.50)	< 0.001
Liver resection time, median[IQR], min	120(71–179)	160(128–231)	0.002
LND time, median[IQR], min	65(57–72)	92(85–99)	< 0.001
Waiting-time, median[IQR], min	29(26.75–32.00)	112(105–119)	< 0.001
Total blood loss, median[IQR], ml	200(150–300)	325(200–500)	0.004
Blood loss during resection, median[IQR], ml	190(138.75–290.00)	295(176.25–480.00)	0.016
Transfusion, n (%)	6(14.3)	28(33.3)	0.023
Conversion, n (%)	0(0)	7(8.3)	0.054
Surgical Bleeding Score, mean ± SD	1.98 ± 1.16	2.37 ± 1.06	0.038
Surgical Bleeding Score, n (%)			0.238
1	17(40.5)	15(17.9)	
2	13(31.0)	35(41.7)	
3	7(16.7)	21(25.0)	
4	4(9.5)	11(13.1)	
5	1(2.4)	2(2.4)	
Surgeon Satisfaction Score, mean ± SD	3.10 ± 0.96	2.52 ± 0.89	0.001
Surgeon Satisfaction Score, n (%)			0.002
1	3(7.1)	11(13.1)	
2	8(19.0)	29(34.5)	
3	13(31.0)	33(39.3)	
4	18(42.9)	11(13.1)	
Vascular reconstruction, n (%)	9(21.4)	16(19.0)	0.752
Lactate level, median[IQR], mmol/L			
Preoperative lactate	0.85(0.70–1.00)	0.80(0.65–0.95)	0.721
Pre-resection lactate	1.10(0.80–1.20)	1.50(1.30–1.70)	< 0.001
Post-resection lactate	1.60(1.50–1.80)	2.10(1.80–2.45)	< 0.001
Postoperative lactate	1.35(1.30–1.40)	1.90(1.50–1.80)	< 0.001
STAI, mean ± SD	11.00(10.00–12.00)	13.00(11.00–17.00)	< 0.001
Margin, n (%)			1.000
R0	41(97.6)	81(96.4)	
R1	1(2.4)	3(3.6)	
Tumor differentiation, n (%)			0.849
Well	1(2.4)	2(2.4)	
Moderate	29(69.0)	62(73.8)	
Poor	12(28.6)	20(23.8)	
Microvascular invasion, n (%)	17(40.5)	38(45.2)	0.611
Capsule, n (%)	5(11.9)	11(13.1)	1.000
Satellite lesion, n (%)	5(11.9)	9(10.7)	1.000
AJCC staging, n (%)			0.797
IA	2(4.8)	3(3.6)	
IB	1(2.4)	4(4.8)	
II	0	1(1.2)	
IIIA	0	0	
IIIB	39(92.9)	76(90.5)	

*LND* lymph nodes dissection, *STAI* State Trait Anxiety Inventory, *AJCC* American Joint Committee on Cancer (8th)

### Quality of liver resection

The optimized group had a significantly lower surgical bleeding score (1.98 ± 1.16 *vs.* 2.37 ± 1.06, *P* = 0.038) and a significantly higher surgeon satisfaction score (3.10 ± 0.96 *vs.* 2.52 ± 0.89, *P* = 0.001; Table 2). Relative to the traditional group, the optimized group also had significantly less blood loss during hepatectomy (190 mL [IQR: 139–290] *vs.* 295 mL [IQR: 176–480], *P* < 0.001), significantly less total blood loss (200 mL [IQR: 150–300] *vs.* 295 mL [IQR: 176.25–480], *P* = 0.016), a lower rate of rate transfusion (14.3% *vs.* 33.3%, *P* = 0.023), and a marginally lower conversion rate (0% *vs.* 8.3%, *P* = 0.054). No patients in the optimized group converted to open surgery. However, 7 patients in the traditional group (8.33%) converted to an open approach due to severe bleeding of the hepatic surgical field during liver resection.

The optimized group had a shorter total operation time (222 min [IQR: 177–297] *vs.* 280 min [IQR: 250–355), *P* < 0.001), shorter “waiting time” (29 min [IQR: 27–32] *vs.* 112 min [IQR: 105–119], *P* < 0.001), shorter liver resection time (119 min [IQR: 79–179] *vs.* 160 min [IQR: 128.5–230], *P* = 0.001), and a shorter LND time (65 min [IQR: 70–85] *vs.* 92 min [IQR: 85–99], *P* < 0.001).

**Fig. 2:**
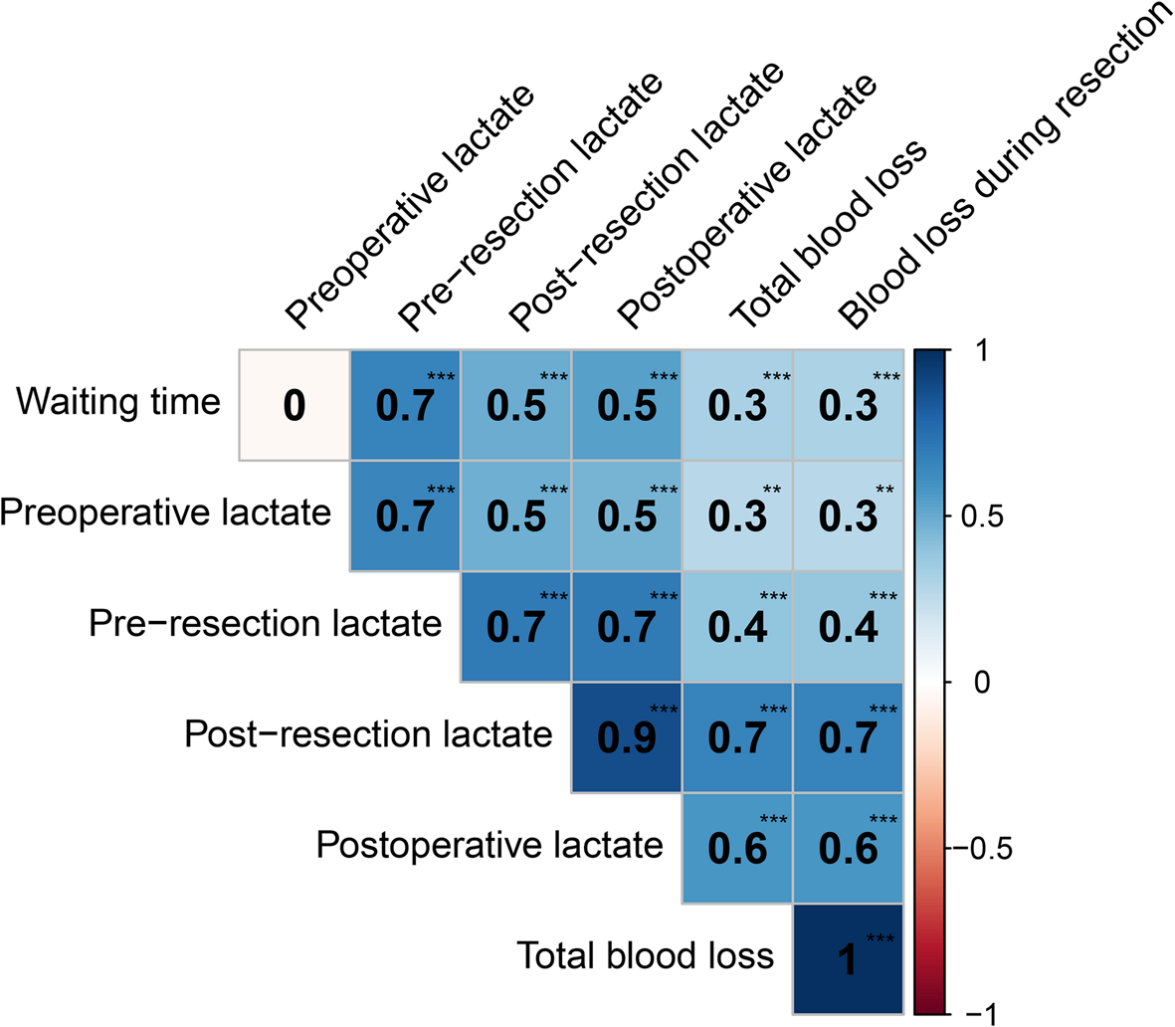
Pearson correlation matrix showing the relationship of “waiting time” with lactate level and blood loss. ***, *P* < 0.001; **, *P* < 0.01

The difference of the preoperative lactate level in the optimized and traditional groups was not statistically significant (*P* = 0.723), but the optimized group had a lower pre-resection lactate level (*P* < 0.001), a lower post-resection lactate level (*P* < 0.001), and a lower postoperative lactate level (*P* < 0.001; Table 2). Pearson correlation analysis showed there were positive correlations of “waiting time” with pre-resection lactate, post-resection lactate, postoperative lactate, total blood loss, and blood loss during liver resection (all *P* < 0.001, Fig. 2). However, the two groups had no significant difference in oncological outcomes (Table 2).

### Quality of LND

The optimized group and traditional group had similar LN positivity (92.9% *vs.* 90.5%, *P* = 0.113; Table 3). However, the optimized group had significantly more harvested LNs (12.0 [IQR: 10.0–13.0] *vs.* 8.0 [IQR: 7.0–9.5], *P* < 0.001) and more positive LNs (8.0 [IQR: 8.0–10.0] *vs.* 5.0 [IQR: 5.0–6.0], *P* < 0.001). Thirty-seven patients (88.1%) in the optimized group achieved adequate LND (6 or more harvested LNs) and 65 patients (77.4%) in the traditional group achieved adequate LND (*P* = 0.149). We also analyzed LN metastasis and adequate LND for each T stage of patients with N1 status (Table 3). The optimized group had more harvested LNs (12.0 [IQR: 10.5–13.5] *vs.* 8.0 [IQR: 7.0–10.0], *P* < 0.001) and more positive LNs (9.0 [IQR: 8.0–10.0] *vs.* 5.5 [IQR: 5.0–6.0], *P* < 0.001), but the two groups were similar in the rate of adequate LND (89.7% *vs.* 77.6%, *P* = 0.111).

**Table 3:** The quality evaluation of lymph node dissection

**Variates**	**Optimized group**	**Traditional group**	***p***
	***n*** ** = 42**	***n*** ** = 84**	
Lymph node metastasis, n (%)			0.113
N1	39 (92.9)	76 (90.5)	
N0	3 (7.1)	8 (9.5)	
N1 distribution, n (%)			0.881
T1A	13 (33.3)	19 (25.0)	
T1B	11 (28.2)	25 (32.9)	
T2	9 (23.1)	17 (22.4)	
T3	4 (10.3)	11 (14.5)	
T4	2 (5.1)	4 (5.3)	
Harvested lymph nodes, median[IQR]	12.0(10.0–13.0)	8.0(7.0–9.5)	< 0.001
Positive lymph nodes, median[IQR]	8.0(8.0–10.0)	5.0(5.0–6.0)	< 0.001
Adequate LND, n (%)	37(88.1)	65(77.4)	0.149
T1A	12(32.4)	17(26.2)	
T1B	10(27.0)	19(29.2)	
T2	9(24.3)	18(27.7)	
T3	4(10.8)	9(13.8)	
T4	2(5.4)	2(3.1)	
**Patient with N1**			
Harvested lymph nodes, median[IQR]	12.0(10.5–13.5)	8.0(7.0–10.0)	< 0.001
Positive lymph nodes, median[IQR]	9.0(8.0–10.0)	5.5(5.0–6.0)	< 0.001
Adequate LND, n (%)	35(89.7)	59(77.6)	0.111
T1A	10(28.6)	15(25.4)	
T1B	10(28.6)	16(27.1)	
T2	9(25.7)	17(28.8)	
T3	4(11.4)	9(15.3)	
T4	2(5.7)	2(3.4)	

*LND* lymph nodes dissection

### Postoperative recovery outcomes

The optimized group had a shorter PLOS (11.33 ± 2.54 *vs.* 13.49 ± 3.85, *P* < 0.001), bowel recovery time (2.38 ± 0.58 *vs.* 2.90 ± 0.79, *P* < 0.001), time before resumption of oral-intake (1.93 ± 0.71 *vs.* 2.63 ± 0.67, *P* < 0.001), and off-bed activity time (2.29 ± 0.71 *vs.* 2.83 ± 0.92, *P* = 0.001, Table 4). The optimized group also had a lower incidence rate of total morbidities (14.3% *vs.* 36.9%, *P* = 0.009), although the groups had no significant differences in specific complications except delirium, which was more common in the traditional group (13.1% *vs.* 2.4%, *P* = 0.046). Multivariate logistic regression analysis showed that the optimized group had a reduced incidence of overall morbidities (OR: 0.332 [95%CI: 0.123–0.891], *P* = 0.029, Table 5).

**Table 4:** Postoperative outcome

**Postoperative outcome**	**Optimized group**	**Traditional group**	***p***
	***n*** ** = 42**	***n*** ** = 84**	
Time to resume, mean ± SD, d			
PLOS	11.33 ± 2.54	13.49 ± 3.85	< 0.001
Off-bed activities	2.29 ± 0.71	2.83 ± 0.92	0.001
Intake	1.93 ± 0.71	2.63 ± 0.67	< 0.001
Bowel movement	2.38 ± 0.58	2.90 ± 0.79	< 0.001
POD1-WBC, mean ± SD, 10^9	9.75 ± 1.21	12.52 ± 1.72	< 0.001
POD3-WBC, mean ± SD, 10^9	7.43 ± 1.18	10.33 ± 1.21	< 0.001
Morbidity, n (%)	6(14.3)	31(36.9)	0.009
Hemorrhage, n (%)	1(2.4)	3(3.6)	1.000
Bileleakage, B class, n (%)	1(2.4)	8(9.5)	0.270
Abdominal abscess,n (%)	3(7.1)	11(13.1)	0.383
Liver failure, n (%)	1(2.4)	2(2.4)	1.000
Septic shock, n (%)	1(2.4)	4(4.8)	0.664
Wound infection, n (%)	1(2.4)	3(3.6)	1.000
Ileus, n (%)	2(4.8)	5(6.0)	1.000
Renal insufficiency, n (%)	1(2.4)	7(8.3)	0.267
Arrhythmia, n (%)	0(0)	3(3.6)	0.550
Delirium, n (%)	1(2.4)	11(13.1)	0.046
Pulmonary infection, n (%)	2(4.8)	9(10.7)	0.334
Death, n (%)	0(0)	2(2.4)	0.552
Reoperation, n (%)	0(0)	0(0)	1.000
Readmission, n (%)	1(2.4)	2(2.4)	1.000
Clavien-Dindo, n (%)			0.206
I	1(2.4)	10(11.9)	
II	1(2.4)	5(6.0)	
III	3(7.1)	9(10.7)	
IV	1(2.4)	5(6.0)	
V	0(0)	2(2.4)	
Lymphadenectasis in initial monitoring, n (%)	5(11.9)	21(25.0)	0.066

*PLOS* postoperative lenth of stay, *POD1-WBC* white blood cell count of postoperative day 1, *POD3-WBC* white blood cell count of postoperative day 3

**Table 5:** The logistic analysis of morbidity

**Variates**	**Univariate**		**Multivariate**	
	**OR(95%CI)**	***p***	**OR(95%CI)**	***p***
Age, ≥ 60y	1.395(0.645–3.016)	0.398		
Sex, male	0.763(0.352–1.653)	0.492		
BMI, ≥ 25	1.554(0.668–3.617)	0.306		
ASA class		0.744		
I	Ref			
II	1.622(0.471–5.589)	0.444		
III	1.441(0.403–5.150)	0.574		
Diabetes mellitus	1.003(0.327–3.078)	0.996		
Hypertension	0.468(0.192–1.144)	0.096		
Previous abdominal surgery	1.420(0.390–5.175)	0.595		
Child–pugh grade, B class	1,706(0.273–10.662)	0.568		
Surgery scope, right hemihepatectomy	1.326(0.595–2.954)	0.490		
Vascular reconstruction	1.372(0.467–4.034)	0.565		
Conversion	6.797(1.255–36.804)	0.026		0.220
Transfusion	3.600(1.562–8.298)	0.003	3.060(1.299–7.208)	0.011
Three-step process optimization	0.285(0.108–0.753)	0.011	0.343(0.126–0.928)	0.035
Waiting-time		0.028		0.287
0-60 min	Ref			
61-120 min	3.200(1.182–8.665)	0.022		
> 120 min	5.250(1.385–19.903)	0.015		
Postoperative lactate		0.018		0.597
1.0–1.6 mmol/L	Ref			
1.7–2.2 mmol/L	2.872(1.198–6.884)	0.018		
> 2.2 mmol/L	3.829(1.240–11.823)	0.020		

*BMI* body mass index, *ASA* American Society of Anesthesiologists

### Survival outcome

**Fig. 3:**
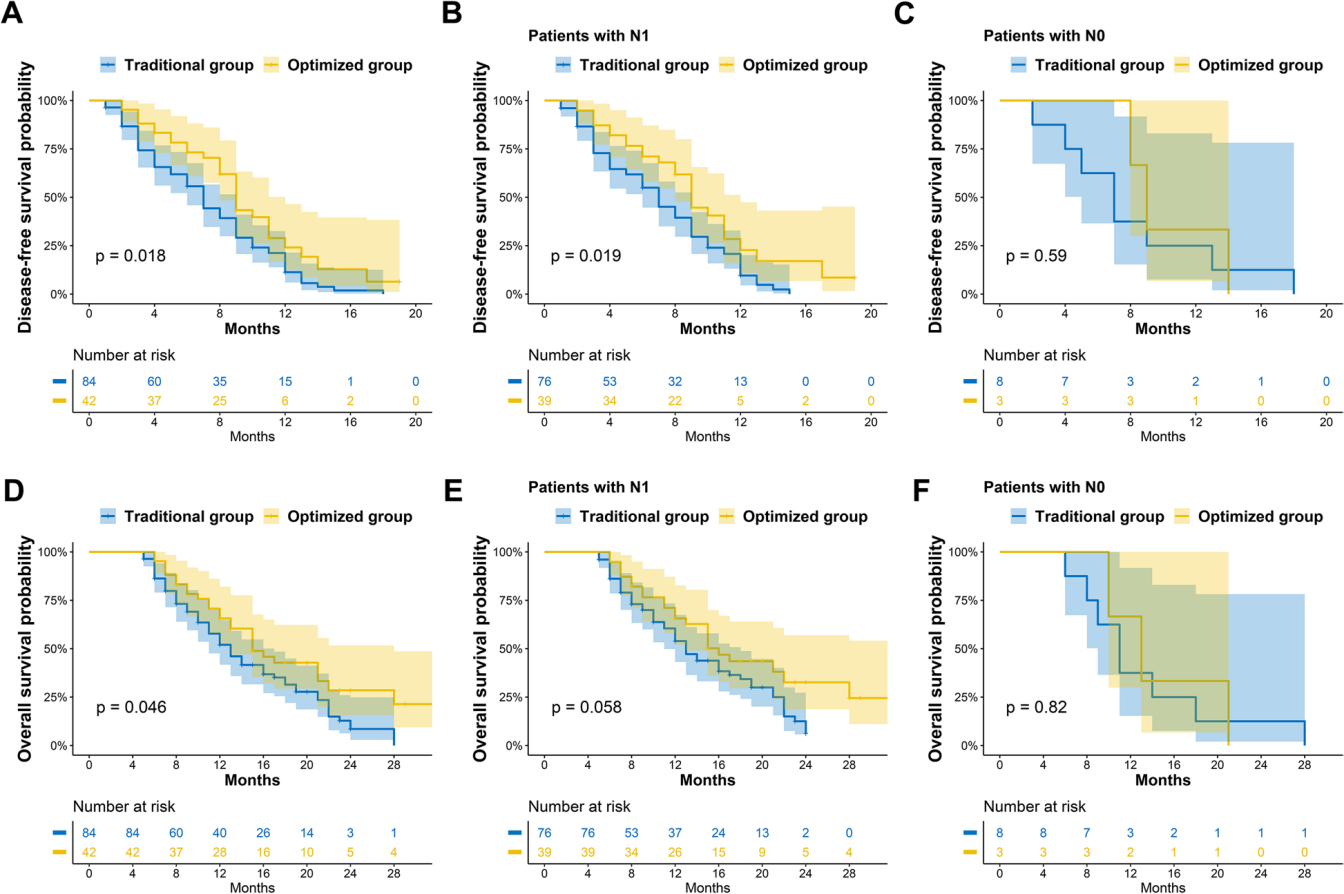
Disease-free survival of all patients in the optimized and traditional groups (**A**), the subgroup with positive lymph nodes (N1) (**B**), and the subgroup with negative lymph nodes (N0) (**C**). Overall survival of all patients in the optimized and traditional groups (**D**), the subgroup with positive lymph nodes (N1) (**E**), and the subgroup with negative lymph nodes (N0) (**F**)

Analysis of DFS showed that the optimized group had a better outcome (9.00 months [IQR: 8.12–9.88] *vs.* 7.00 months [IQR: 6.05–8.00], *P* = 0.018, Fig. 3A). The results were similar for patients with stage N1 (*P* = 0.019, Fig. 3B). Patients in the two groups who had stage N0 had no significant difference in DFS (*P* = 0.590, Fig. 3C). The optimized group also had a significantly longer OS (15.0 months [IQR: 11.39–18.62] *vs.* 13.00 months [IQR: 10.87–15.14], *P* = 0.046, Fig. 3D–F).

Cox regression analysis confirmed the optimized three-step procedure provided a better DFS (OR: 0.627 [95%CI: 0.407–0.967], *P* = 0.035, Table 6) and marginally better OS **(**OR: 0.625 [95%CI: 0.408–1.043], *P* = 0.074, Table 6). Notably, the imaging results from the initial postoperative monitoring also showed there was a tendency for a greater prevalence of signs of lymphadenectasis around the hepatoduodenal ligament in the traditional group than in the optimized group (25.0% *vs.* 11.9%, *P* = 0.066).

**Table 6:** The cox analysis of DFS

	**Univariate for DFS**		**Multivariate for DFS**		**Univariate for OS**		**Univariate for S**	
	**OR(95%CI)**	***p***	**OR(95%CI)**	***p***	**OR(95%CI)**	***p***	**OR(95%CI)**	***p***
Age, ≥ 60y	0.957(0.623–1.471)	0.841			0.932(0.575–1.512)	0.932		
Sex, male	0.897(0.587–1.370)	0.614			1.001(0.637–1.574)	0.996		
Tumor location, right side	0.735(0.467–1.156)	0.183			0.708(0.435–1.151)	0.163		
Tumor number, multiple	0.782(0.308–1.987)	0.606			0.589(0.191–1.814)	0.357		
Three-step process optimization	0.640(0.403–1.017)	0.059	0.627(0.407–0.967)	0.035	0.640(0.398–1.031)	0.066	0.652(0.408–1.043)	0.074
T stage		0.879				0.845		
T1A	Ref				Ref			
T1B	1.198(0.697–2.060)	0.514			1.169(0.670–2.041)	0.582		
T2	1.242(0.701–2.203)	0.458			1.119(0.593–2.111)	0.729		
T3	1.307(0.501–2.144)	0.922			0.947(0.427–2.100)	0.893		
T4	1.720(0.481–6.146)	0.404			1.932(0.555–6.724)	0.301		
Margin, R1	0.535(0.154–1.855)	0.324			3.059(0.868–10.779)	0.082	2.250(0.690–7.341)	0.179
Tumor differentiation		0.059				0.012		0.014
Well	Ref		Ref		Ref		Ref	
Moderate	0.870(0.533–1.418)	0.575	0.915(0.585–1.431)	0.696	0.844(0.503–1.417)	0.522	0.806(0.493–1.318)	0.390
Poor	4.009(1.075–14.952)	0.039	3.452(1.009–11.805)	0.048	6.117(1.575–23.763)	0.009	4.793(1.374–16.725)	0.014
Macrovascular invasion	0.973(0.502–1.883)	0.934			0.723(0.339–1.542)	0.401		
Microvascular invasion	1.547(0.981–2.440)	0.060	1.472(0.978–2.215)	0.064	1.548(0.955–2.511)	0.076	1.395(0.889–2.189)	0.147
Capsule	0.970(0.527–1.788)	0.923			1.073(0.551–2.090)	0.835		
Satellite lesion	0.722(0.345–1.511)	0.387			0.475(0.205–1.103)	0.083	0.520(0.236–1.144)	0.104

*DFS* disease-free survival, *OS* overall survival, *LND* lymph nodes dissection

## Discussion

Many studies advocated the use of laparoscopy for surgical treatment of ICC because this technique is minimally invasive, allows abdominal exploration, improves early recovery, and provides a non-inferior oncological survival benefit [19, 36, 41–46]. However, this procedure can be technically difficult, complicated, require a long operation time, and is less developed, especially for patients with advanced-stage ICC [10, 19, 36, 41–46]. To the best of our knowledge, this study is the first to propose an optimization of the laparoscopy procedure for advanced ICC, which was designed to improve the quality of liver resection and LND, increase the duration of DFS, shorten the operation time, and improve postoperative recovery. In contrast to many previous studies of this topic, we adopted a rigorous study design. First, all enrolled patients had advanced ICC with high probabilities of preoperative LN metastasis and all patients received regional LND. This reduced the outcome bias caused by the incomplete or lack of LND of some patients, as reported in previous studies [7, 16, 45]. Second, we used PS matching to compare the two groups, and accurately determined the blood loss, the times needed for liver resection and LND, and the quality of liver resection and LND. Our comprehensive and detailed examination of these procedures from multiple angles helped to reduce selection bias and improve the reliability of the results. Finally, this study was the first to consider the variable of “waiting-time” during the process of liver resection.

Unlike treatments for HCC, treatments for ICC require hepatoduodenal ligament LND. This procedure can be time-consuming when using a laparoscopic approach, and increases the “waiting time” during LCVP, possibly having an adverse effect on the quality of subsequent liver resection. We found a positive correlation between “waiting time” and blood loss (Fig. 2), a result not reported in previous studies. This correlation may be due to the accumulation of acidic products and local vasodilators in the liver caused by long-term LCVP. During relative hypoxia, production of catecholamines, and the massive accumulation of lactic acid, 5-HT, adenosine, and bradykinin substances can occur. This can lead to microvascular expansion, vascular reactivity, and decreased contractility, and result a decreased quality of liver resection and increased bleeding [47–49]. The higher lactate levels in our traditional group before and after liver resection reflected this difference. Many studies reported that a laparoscopic approach can reduce blood loss compared with open surgery. The blood loss in our traditional group was similar to that reported in some studies, but lower than that from open surgery in other studies [10, 18, 20, 44, 50–54]. The present study is the first to report that optimizing the laparoscopic procedure can reduce blood loss and improve the condition of the hepatic surgical field. A dry surgical field in the liver provides better visualization of the intrahepatic vessels, and therefore reduces bleeding and conversion caused by accidental injury to these intrahepatic vessels [47–49]. When the surgical field is in a better condition, this reduces the rate of transfusion and conversion, and the time needed for hepatectomy, leading to a shorter operation time and improved intraoperative outcomes [20, 46, 51–54].

Surgeons increasingly prefer routine regional LND, and the AJCC recommends harvesting more than 6 LNs [12]. However, there is limited understanding of quality of LND using a laparoscopic approach [4, 36, 50, 55]. We unexpectedly found that optimizing the surgical procedure improved the quality of laparoscopic LND and increased the number of harvested LNs (10.0 *vs.* 8.0, *P* < 0.001). Furthermore, the rate of adequate LND in this study reached 88.1% (optimized procedure) and 77.4% (traditional procedure), higher than the 20 to 43% reported in previous research [55]. A recent multi-center study of ICC by Brustia et al. showed that laparoscopic surgery was non-inferior to open surgery in terms of OS and DFS, and led to similar numbers of harvested LNs (2.23 ± 0.78 *vs.* 2.42 ± 1.25), but their corresponding rates of performed LND was only 17.9% (laparoscopic) and 21.3% (open) [44]. Another multi-center study showed that laparoscopic surgery led to a greater number of harvested LNs (5 [IQR: 4–7] *vs.* 3 [IQR: 2–6]) and a higher rate of adequate LND (43% *vs.* 35%), but these differences were not statistically significant and may have been biased by the different numbers of patients who received LND [10].

Compared with previous studies, we harvested more LNs and had a higher rate of adequate LND. We can suggest several reasons for these results. First, we only included patients with advanced-stage ICC, all of whom received LND because of preoperative suspicious LNs. Second, our center is the largest hepato-biliary-pancreatic center in Southeast China, and has extensive experience in laparoscopic liver resection and LND [56, 57]. Third, at a technical level, removal of the lesion improves the visual field when there is no occlusion by LNs. In the traditional procedure, one of the arms of the assistant’s forceps must be used to help expose the hepatic duodenal ligament area from occlusion by the liver. This procedure is unnecessary when using the optimized procedure, and this allows experienced surgeons at each side (a “bilateral two chief surgeons approach”) for exposing the hilar blood vessels and performing the LND [57]. Fourth, the main task (liver resection) in the optimized group was completed before the LND, and there was almost no psychological pressure on the surgical team due to the “waiting time”. A more relaxed mental state is conducive to more refined operations during surgery [58–60], and the lower STAI in the optimized group reflects this benefit. Moreover, the presence of more positive LNs in the optimized group may be because this group had more harvested LNs; the two groups had no significant difference in the percentages of positive LNs.

Interestingly, our observations indicated that some surgeons in the traditional group were eager to complete the LND first. As a result, the quality of some LNDs was unsatisfactory. Some surgeons who had more patience may spend a long time in carefully performing LND and achieve high-quality results. However, this could lead to an excessive “waiting time”, so that the subsequent condition of the hepatic surgical field was unsatisfactory, often with large blood loss and the need for an extremely long operation time. In other words, using the traditional procedure does not allow both high-quality LND and a short “waiting time”. The optimization procedure described here resolves this dilemma. A more encouraging result is our finding of longer median DFS and OS in the optimized group. This may be because of the improved quality of LND and the earlier discharge and receipt of chemotherapy [61]. Subgroup analysis and Cox regression analysis confirmed these findings (Fig. 3, Table 6). Among patients with stage N0, our two groups had no significant difference in DFS and OS, consistent with previous studies [4, 13].

Patients in our optimized group also had a lower incidence of total morbidities and a faster postoperative recovery, possibly because of the shorter operation time and lower blood lactate levels. Previous studies reported that an excessive operation time increased the risk of intraoperative blood loss, lactic acid level, and various perioperative adverse events [27]. Optimizing the surgical procedure and shortening the operation time can also reduce the frequency of adverse events [62]. Our regression analysis confirmed that optimizing the surgical procedure reduced total morbidities. The incidence rates of complications in our two groups were similar to those reported in previous studies, indicating that the optimized three-step procedure described here is safe and feasible [41, 44–46]. Importantly, the optimized procedure described here is also low-cost, easy to develop, and provides good clinical value.

This study had some limitations. First, this was an observational study. Although selection bias cannot be completely prevented, we used PS matching to minimize this bias. Second, this study was performed at a single center in China, and therefore requires confirmation by studies at other centers. Third, given the small sample size, further follow-up of survival times is required in the future.

## Conclusions

Based on the concept of “waiting time”, we developed a three-step optimized laparoscopic procedure for advanced ICC to improve the quality of liver resection and LND, to prolong DFS and OS, and to provide better intraoperative and postoperative outcomes. Our results indicated the three-step laparoscopic procedure described here is feasible and effective, and should be considered for patients with advanced ICC.

## Data Availability

All data generated during this study are shown in the figures and tables. The datasets generated during the current study are not publicly available, but are available from the corresponding author on reasonable request.
